# Triple-negative pleomorphic lobular carcinoma and expression of androgen receptor: Personal case series and review of the literature

**DOI:** 10.1371/journal.pone.0235790

**Published:** 2020-07-22

**Authors:** Kohei Taniguchi, Shinichi Takada, Masako Omori, Takuro Igawa, Midori Filiz Nishimura, Toshiaki Morito, Kouichi Ichimura, Tadashi Yoshino

**Affiliations:** 1 Department of Pathology, Okayama University Hospital, Okayama, Japan; 2 Department of Pathology, Yuai Memorial Hospital, Koga, Ibaraki, Japan; 3 Department of Pathology, Kurashiki Medical Center, Kurashiki, Japan; 4 Department of Pathology, Okayama University Graduate School of Medicine, Dentistry and Pharmaceutical Science, Okayama, Japan; 5 Department of Pathology, National Hospital Organization Iwakuni Clinical Center, Iwakuni, Japan; 6 Department of Pathology, Hiroshima City Hiroshima Citizens Hospital, Hiroshima, Japan; Universita degli Studi di Torino, ITALY

## Abstract

Pleomorphic lobular carcinoma (PLC) is a histological variant of invasive lobular carcinoma (ILC) and is associated with worse prognosis than classical ILC. It exhibits a greater degree of cellular atypia and pleomorphism and is occasionally accompanied with apocrine morphology. We investigated the immunohistochemical characteristics of samples from 31 Japanese patients with PLC to elucidate the clinicopathological characteristics of PLC including androgen receptor (AR) immunoreactivity. The surrogate molecular subtypes were luminal A-like, luminal B-like, luminal B-like/HER2, HER2-type, and triple-negative in 5, 4, 3, 5, and 14 cases, respectively. AR was positive in 92.8% (13/14) of the triple-negative PLC cases and 100% (10/10) of the non-triple-negative PLC cases. Disease-specific survival was worse in patients with histological grade 3 PLCs than in those with histological grade 2 PLCs (*p* = 0.007). However, there was no significant difference in the progression-free survival between the two groups (*p* = 0.152). No other clinicopathological characteristics were associated with prognosis. These results reveal that PLC exhibits various surrogate molecular subtypes and that the triple-negative subtype frequently expresses AR. The observed molecular apocrine differentiation implicates that triple-negative PLC can be categorized into the luminal AR subtype. Furthermore, AR-targeted therapy might be useful for patients with triple-negative PLC.

## Introduction

Invasive lobular carcinoma (ILC) comprises 10%–15% of all breast cancers [1]. Currently, the World Health Organization defines ILC as an invasive carcinoma composed of dyscohesive cells individually dispersed or arranged in a single-file linear pattern in a fibrous stroma [2]. Typical ILCs infrequently exhibit nuclear atypia and mitoses. ILC is characterized by the reduced expression of *CDH1* that encodes E-cadherin [3]. Loss of E-cadherin is detected via immunohistochemistry in approximately 90% of ILCs [1–3]. Conversely, most ILCs are frequently positive for estrogen receptor (ER) and progesterone receptor (PgR), whereas they are frequently negative for human epidermal growth factor receptor 2 (HER2) [1, 2]. Furthermore, *HER2* amplification is rarely detected in ILC via fluorescent *in situ* hybridization.

Pleomorphic lobular carcinoma (PLC) is a rare histological variant of ILC [4–7]. Although retaining the characteristic growth pattern of ILC, PLC exhibits more significant cellular atypia and pleomorphism compared with the classical ILC [5, 6]. In addition, several studies have reported apocrine differentiation in PLC [6–8]. Most PLC cases exhibit PLC *in situ* (PLCIS) surrounding the invasive component [9]. In contrast to the classical ILC, PLC tends to be HER2-positive as detected by immunohistochemistry [8]. Further, the overall survival of patients with PLC appears to be inferior to that of patients with classical ILC [4].

Triple-negative breast cancer (TNBC) is a heterogeneous disease characterized by the lack of expression of ER, PgR, and HER2. TNBC can be genetically classified into four subtypes: basal-like including BL1 and BL2, mesenchymal, and luminal androgen receptor (LAR). The LAR subtype is characterized by augmented androgen receptor (AR) signaling [10, 11]. Several studies have developed specific molecular classifications of TNBC, including the LAR subtype [11–13]. One study reported that the pathologic complete response was worst in patients with LAR subtype of TNBC among all TNBC molecular subtypes [13]. Interestingly, apocrine carcinoma, a special type of breast cancer, typically exhibits the immunohistochemical profile of TNBC with AR positivity [14]. These findings suggest that apocrine carcinoma may be closely related to the LAR subtype of TNBC. Moreover, some researchers recommend that morphological and IHC criteria (apocrine morphology in >90% of cells together with ER and PR negativity and AR positivity in at least 10% of tumor cell nuclei) should be used to define apocrine carcinoma [15]. AR-positive TNBC, referred to as molecular apocrine breast cancer, typically arises in older women and has a relatively better prognosis than AR-negative TNBC [14, 16–19]. Although PLCs apocrine features have been reported, previous studies have not confirmed AR positivity in triple-negative PLC [6–8, 20, 21].

This study aims to describe the clinicopathological findings of 31 patients with invasive PLC in Japan with review of the literature including AR immunoreactivity.

## Materials and methods

### Case selection

In this study, we enrolled 31 Japanese patients with invasive PLC, including 22 patients from Hiroshima City Hiroshima Citizens Hospital and 9 patients from Okayama University Hospital, who were diagnosed between 2012 and 2018. Histologic diagnoses were based on the 5^th^ edition of the World Health Organization classification [2]. All cases were reviewed by two pathologists (S.T. and K.T. for cases from Hiroshima City Hiroshima Citizens Hospital, and M.O. and K.T. for cases from Okayama University Hospital) to confirm PLC morphology, such as the presence of large nuclei, prominent nucleoli, and pleomorphism. Additionally, E-cadherin negativity was confirmed via immunohistochemistry. The follow-up data were available for all cases. The use of patient specimens and medical records was approved by the Institutional Review Board of Okayama University, Japan (IRB approval number:1906–004). The consent was not obtained because of lost follow up or death from the disease. Instead of informed consent, we use opt out on the website. The IRB of Okayama University have approved this method. The study has been performed in accordance with the ethical standards laid down in the Declaration of Helsinki. All data were fully anonymized after we accessed them. From 2015 to 2016, the medical records of 22 patients from Hiroshima City Hiroshima Citizens Hospital were accessed, and in 2018, those of 9 patients from Okayama University Hospital were accessed.

### Histological analyses

Tissue specimens were fixed with 10% formalin and embedded in paraffin. Sections, 4 μm in thickness, were stained with hematoxylin-eosin. Histological components, such as ductal carcinoma *in situ*, lobular carcinoma *in situ*, PLCIS, invasive carcinoma of no special type (NST), and ILC, were recorded. Histological grading was based on the Nottingham histological grading system, as previously reported [22].

### Immunohistochemical analyses

Immunostaining was performed using formalin-fixed, 4-μm, paraffin-embedded tissue sections with the Ventana BenchMark ULTRA system (Roche Diagnostics, Basel, Switzerland) according to the manufacturer’s instructions. The slides were treated with a deparaffinization solution followed by epitope retrieval. The list of primary antibodies used in the study is presented in Table 1. Localization of the antigen-antibody complex was achieved using the OptiView Universal DAB Detection Kit (Roche Diagnostics, Basel, Switzerland) for the 22 cases from Hiroshima City Hiroshima Citizens Hospital and using the ultraView Universal DAB Detection Kit (Roche Diagnostics, Basel, Switzerland) for the 9 cases from Okayama University Hospital.

**Table 1:** List of the immunohistochemical antibodies.

Antibody	Clone	Dilution	Distributor	Hospital
ER	SP1	Prediluted	Roche Diagnostics, Basel, Switzerland	OUH, HCH
		Ready to use		
PgR	1E2	Prediluted	Roche Diagnostics, Basel, Switzerland	OUH, HCH
		Ready to use		
HER2	4B5	Prediluted	Roche Diagnostics, Basel, Switzerland	OUH, HCH
		Ready to use		
Ki-67	MIB-1	1:50	Agilent technologies, Santa Clara, United States.	OUH
	30–9	Prediluted	Roche Diagnostics, Basel, Switzerland	HCH
		Ready to use		
AR	SP107	Prediluted	Roche Diagnostics, Basel, Switzerland	OUH, HCH
		Ready to use		
GCDFP-15	23A3	1:40	Leica Microsystems, Wetzlar, Germany	OUH
		1:50	Agilent technologies, Santa Clara, United States.	HCH
E-cadherin	NCH-38	Prediluted	Nichirei Bioscience, Tokyo, Japan	OUH
		Ready to use		
		Prediluted	Roche Diagnostics, Basel, Switzerland	HCH
		Ready to use		

AR, androgen receptor; ER, estrogen receptor; GCDFP-15, gross cystic disease fluid protein-15; HCH, Hiroshima City Hiroshima Citizens Hospital; HER2, human epidermal growth factor receptor type 2; OUH, Okayama University Hospital; PgR, progesterone receptor; PLC, pleomorphic lobular carcinoma

The ER and PgR status was based on the following current definitions: positive, ≥1% nuclear staining and negative, <1% nuclear staining [23]. The HER2 status was determined according to the American Society of Clinical Oncology/College of American Pathologists guidelines [24]. AR immunoreactivity was considered positive in cases with 10% or more tumor cells that were positive, according to a previous study [25]. The tumor was considered positive for gross cystic disease fluid protein-15 (GCDFP-15) in the presence of immunoreactivity in any of the tumor cells.

The invasive PLC cases were categorized into five subtypes based on their immunohistochemical profiles: luminal A-like, luminal B-like, luminal B-like/HER2, HER2-type, and triple-negative, as defined previously [26]. The cases were categorized as high or low in Ki-67 labeling index (LI) using the cutoff value of 14% according to the St.Gallen International Expert Consensus [26].

### Statistical analysis

To compare disease-specific survival (DSS) and progression-free survival (PFS), survival curves were generated using the Kaplan–Meier method and analyzed using the log-rank test with SPSS version 14.0 J (IBM, Armonk, NY). A P value of <0.05 was considered to indicate statistical significance. DSS was defined as the time from the diagnosis of breast cancer until the date of death from the disease. PFS was defined as the time from the diagnosis of breast cancer until the date of first recurrence or progression of the disease or the date of death from any cause. Patients who were not reported to be dead at the time of the analysis were censored at the date when they were last known to be alive.

## Results

### Clinicopathological characteristics

The clinicopathological findings of the patients are summarized in Table 2 (for more details in the S1 File). The median patient age was 66 (range, 40‒86) years. There were 11, 13, 4, and 3 patients with T1, T2, T3, and T4 cancer; the majority of the patients presented with an early T-stage cancer. Among the patients with T1 PLC, 2, 2, and 7 patients were in the T1a, T1b, and T1c stages, respectively. Additionally, 14 patients had lymph node metastases, including 1, 4, 5, and 4 patients in the pNmi, pN1, pN2, and pN3 stages, respectively. Only one patient exhibited distant metastasis. The proportions of the clinical stages varied in the study cohort; the disease stages were IA, IIA, IIB, IIIA, IIIB, IIIC, and IV in 10, 6, 4, 3, 2, 4, and 1 patient, respectively.

**Table 2:** Clinicopathological characteristics of patients with invasive PLC.

Factor			Invasive PLC (n = 31)
Sex			
	Male		0
	Female		31
Age			
	Range (median)		40–86 (66)
Clinical stage			
	1		10
	2		10
	3		9
	4		1
Histological grade		1	0
		2	23
		3	8
	Glandular formation	≤2	0
		3	31
	Nuclear atypia	≤2	0
		3	31
	Mitosis	≤2	27
		3	4
Other histological components			
	Invasive carcinoma of NST		1
	DCIS		2
	Apocrine DCIS		1
	ILC		3
	PLCIS		1
	LCIS		2
Prognosis			
	Follow-up period Range(median)		3.7–113.9 (20.7)
	Survival		26
	Death		5

DCIS, ductal carcinoma in situ; ILC, invasive lobular carcinoma; LCIS, lobular carcinoma in situ; PLC, pleomorphic lobular carcinoma; PLCIS, pleomorphic lobular carcinoma in situ

**Fig 1:**
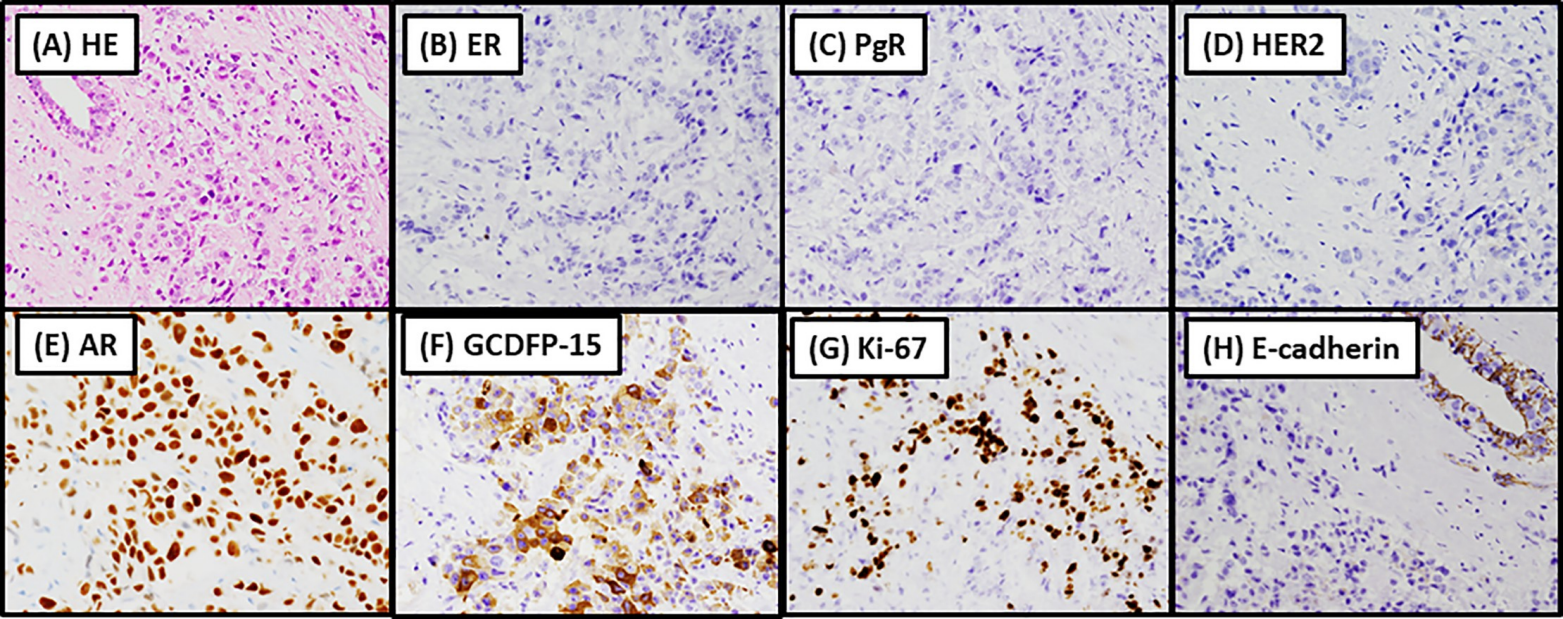
Representative histological and immunohistochemical features of invasive pleomorphic lobular carcinoma. (A) Hematoxylin-eosin staining showing dyscohesive tumor cells with prominent nucleoli and voluminous cytoplasm. Tumor cells are negative for estrogen receptor (ER) (B) and progesterone receptor (PgR) (C) and human epidermal growth factor receptor 2 (HER2) (D) and are positive for androgen receptor (AR) (E) and gross cystic disease fluid protein 15 (GCDFP-15) (F). The Ki-67 labeling index is 30%–50% (G). These findings demonstrate that the tumor is a triple-negative carcinoma with apocrine differentiation. The tumor cells are negative for E-cadherin (H).

The representative histological features of invasive PLC are shown in Fig 1. The tumor cells were dyscohesive with prominent nucleoli and voluminous cytoplasm. Among a total of 31 invasive PLC cases, 23 and 8 cases were histological grades 2 and 3, respectively. Both the glandular formation and nuclear atypia scores were 3 in all 31 cases. The mitotic scores were 1, 2, and 3 in 23, 4, and 4 cases, respectively. Concurrent tumors were found in nine patients, including two patients with ductal carcinoma *in situ*, one patient with apocrine ductal carcinoma *in situ*, one patient with invasive carcinoma of NST, two patients with lobular carcinoma *in situ*, one patient with PLCIS, and three patients with ILC. One patient had two concurrent tumors, ILC and DCIS.

### Immunohistochemistry

The results of immunohistochemical analyses are summarized in Table 3. The representative images of immunohistochemical staining in invasive PLC are shown in Fig 1. The rates of positivity for ER, PgR, and HER2 were 38.7% (12/31), 12.9% (4/31), and 25.8% (8/31), respectively. The Ki-67 LI was high in 13 cases and low in 15 cases with the following distribution of the KI-67 LI: 1%‒13%, n = 15; 14%‒30%, n = 7; 30%‒50%, n = 5; 50%‒80%, n = 1; not available, n = 3. The PLC subtypes of the cohort included luminal A-like, luminal B-like, luminal B-like /HER2, HER2-type, and triple-negative in 5, 4, 3, 5, and 14 cases, respectively (Table 3).

**Table 3:** Molecular subtypes of invasive PLCs.

		Luminal A-like	Luminal B-like	Luminal B-like /HER2	HER2 type	Triple-negative
PLC		5	4	3	5	14
AR	positive	4	3	2	1	13
	negative	0	0	0	0	1
GCDFP15	positive	3	2	2	1	11
	negative	1	1	1	1	0

AR, androgen receptor; GCDFP-15, gross cystic disease fluid protein 15; HER2, human epidermal growth factor receptor type 2, LAR, luminal androgen receptor; PLC, pleomorphic lobular carcinoma

Immunohistochemical analyses of AR and GCDFP-15 was performed in 24 and 23 PLC cases, respectively (Table 3). In total, 92.8% of the triple-negative PLC cases (13/14) and 100% (10/10) of the non-triple-negative PLC cases were positive for AR. Additionally, 100% (11/11) of the triple-negative PLC cases were positive for GCDFP-15 whereas 66.6% (8/12) of the non-triple-negative PLC cases were positive for GCDFP-15.

### Clinical outcomes

The median follow-up time was 20.7 (range, 3.7–113.9) months. Five patients (16.1%) with invasive PLC experienced disease-specific mortality (Table 2), including four patients with triple-negative PLC and one patient with HER2-type PLC.

**Fig 2:**
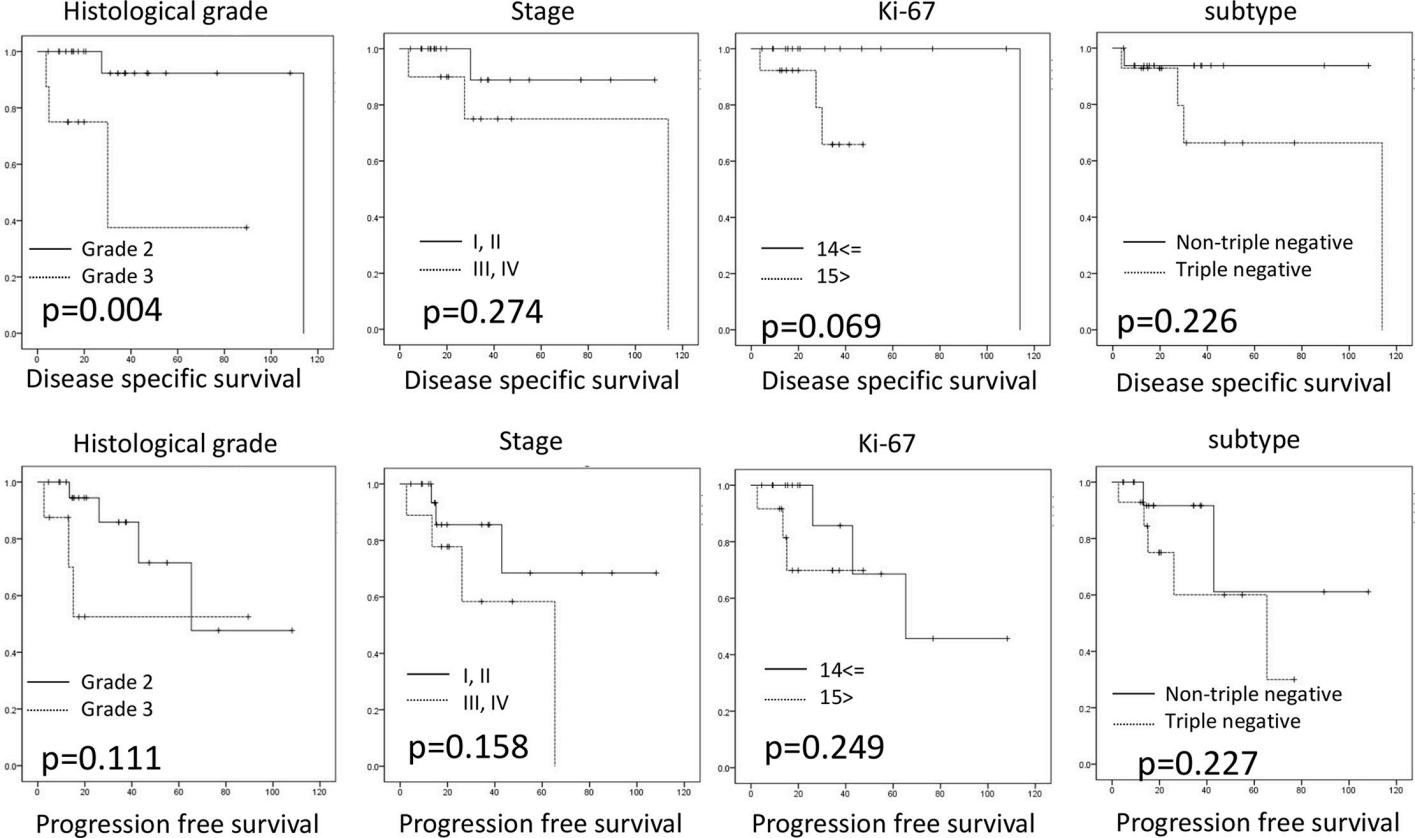
Survival analysis of invasive PLC patients with invasive pleomorphic lobular carcinoma. Disease-specific survival (DSS, A–D) and progression-free survival (PFS, E–H) analyses. The DSS was worse in patients with histological grade 3 cancer than in those with histological grade 2 cancer, although there was no significant difference in the PFS rates between the two groups. Other factors, including clinical stage, high Ki-67 labeling index, and non-triple-negative subtype were not related to DSS or PFS.

The DSS and PFS rates of patients with PLC are shown in Fig 2. Briefly, the DSS of the patients with histological grade 3 PLC was poorer than that of the patients with histological grade 2 PLC (*P* = 0.004), although there was no significant difference in the PFS between the two groups (*P* = 0.111). Clinical stage, high Ki-67 LI, and non-triple-negative subtype were not significant prognostic factors (DSS, *P* = 0.274, 0.069, and 0.226 and PFS, *P* = 0.158, 0.249, and 0.227, respectively).

## Discussion

PLC is a distinct subtype of ILC that is characterized by significant cellular atypia and pleomorphism. In addition, PLCs occasionally exhibit apocrine morphology [2]. Histiocytoid ILCs also exhibit apocrine morphology [2]. In the WHO 5th edition, histiocytoid ILCs are defined as tumor cells with pale eosinophilic cytoplasm and low- to intermediate-grade nuclei, resembling histiocytes [2]. In the present study, all PLC cases were of high histological grade (grades 2 or 3) with the highest score of 3 for nuclear atypia. Hence, our study did not include histiocytoid ILC. In this small cohort, DSS was better in patients with histological grade 2 PLC than in those with histological grade 3 via univariate analysis. Patients with histological grade 2 PLC had a mitotic score of 1 but exhibited conspicuous nuclear atypia. As tumor cells with apocrine differentiation often exhibit large nuclei and prominent nucleoli, the nuclear atypia observed in these cases with histological grade 2 PLC might reflect apocrine differentiation rather than aggressiveness. Therefore, mitotic score can offer useful information in predicting the prognosis of patients with PLC.

It remains controversial whether patients with PLCs have worse prognosis than those with other invasive breast cancers. Previous reports have demonstrated that the prognosis of invasive PLC is significantly worse than that of invasive carcinoma of NST [6, 7, 27, 28]. In contrast, another report has illustrated that PLC is not a prognostic factor compared with invasive carcinoma of NST via multivariate analysis [29]. In the present study, only five patients with PLC died of the disease, and it is unlikely that patients with PLC have worse prognosis than those with other types of breast cancer. However, this result might be due to the relatively early tumor stage at presentation.

The surrogate molecular subtypes of breast cancer based on immunohistochemistry include luminal A-like, luminal B-like, luminal B-like/HER2-positive, HER2-type, and triple-negative [30]. This classification provides critical information necessary for hormone and anti-HER2 therapies [31, 32]. Patients with PLC are more likely to be ER-negative and HER2-positive than those with classical ILC [4, 33, 34]. Accordingly, the PLC cases in the present study included luminal A-like (n = 5), luminal B-like (n = 4), triple-negative (n = 14) and 8 HER2-positive cases comprising luminal B-like /HER2 (n = 3) and HER2-type (n = 5). At present, there are no established standard therapies for PLC, and treatment is based on the surrogate molecular subtypes.

AR positivity is considered essential for demonstrating apocrine differentiation in TNBC [14, 18, 19]. Several studies have reported AR positivity rates in patients with triple-negative invasive carcinoma of NST (Table 4) [35–38]. These studies have utilized different anti-AR antibody clones and different cutoffs for AR positivity. The AR positivity rate in triple-negative PLC cases in the present study (92.8%) was clearly higher than that previously reported in triple-negative invasive carcinoma of NST, which ranged from 17.7% to 41% [35–39]. This characteristic, which highlights the apocrine features, indicates that triple-negative PLC can be classified as the LAR subtype. In a previous report, gene expression analyses described 4 cases of pleomorphic lobular carcinomas as pertaining to the molecular apocrine subgroup or HER2-positive group [20]. Among the 31 PLC cases in the present study, however, there were various surrogate molecular subtypes other than the LAR subtype and HER2-type. Since surrogate classification via IHC is not perfectly correlated with gene expression analyses, further investigations with a larger sample size should be warranted to clarify the gene expression profile of PLC.

**Table 4:** Published studies on androgen receptor immunoreactivity in triple-negative breast cancer.

Reference	Number of cases	AR-positive cases (%)	Clone	Cutoff (%)
Present study	13	92.8%	SP107	10%
Doberstein et al, 2014 [35]	52	40.4%	SP107	10%
Astvatsaturyan et al, 2018 [36]	135	41%	F39.4.1	1%
Choi et al, 2015 [37]	492	17.7%	ER179(2)	1%
Gasparini et al. 2014 [38]	396	24.8%	F.39.4.1	5%

AR, androgen receptor

Several studies have reported that AR positivity is associated with a lower risk of recurrence; on the other hand, a study reported worse outcomes in AR-positive TNBC [36–38, 40]. However, other studies have not found differences in the recurrence risk between AR-positive and AR-negative TNBC [39, 41, 42]. Although the prognostic value of AR immunoreactivity in TNBC remains controversial, preclinical and clinical data have revealed that patients with ER-negative/AR-positive breast cancer might be candidates for treatment with AR antagonist [43]. We found that most of the triple-negative PLCs were AR-positive, which implicates a crucial role for AR signaling in triple-negative PLCs. Therefore, patients with triple-negative PLC might benefit from treatment with AR antagonist. However, the prognosis could not be compared between the AR-positive and AR-negative PLC cases as the cohort included only one AR-negative PLC case; this comparison should be investigated in further studies.

Several studies have previously illustrated the presence of apocrine differentiation in PLC using GCDFP-15 [6, 44]. Accordingly, all triple-negative PLC cases (100%) were positive for GCDFP-15. In addition to the triple-negative PLC cases, all 10 non-triple-negative PLC cases exhibited AR positivity. Relatedly, nine of these ten non-triple-negative PLC cases were ER-positive. In agreement with this finding, ER-positive breast cancers have been demonstrated to frequently express AR and ER [45]. The co-expression of ER and AR have been extensively studied in the literature. In particular studies by Castellano et al. have shown that AR is expressed in 70.9% of ER positive breast cancers [46] and that the AR/ER ratio ≥2 identifies a subgroup of patients with aggressive biological features and may represent an additional independent marker of worse prognosis [47].

Despite the rarity of PLC, there are several case series on invasive PLC (Table 5). One case series included 401 cases of PLCs [4]. However, this case series did not use AR and GCDFP-15 to confirm apocrine features [4]. Although other researchers have investigated AR positivity in invasive PLCs, they have not mentioned whether these invasive PLCs were triple-negative [20, 21]. Moreover, surrogate molecular subtypes were not considered in all the previous reports [4, 6–8, 20, 28, 33, 48]. To the best of our knowledge, this is the first study of a rare case series of invasive PLC, which includes various immunohistochemical data and surrogate molecular subtypes.

**Table 5:** Published studies on invasive PLC case series.

Reference	Number of cases	Immunohistochemistry		Analysis of surrogate molecular subtypes
		AR-positive (%)	GCDFP-15 positive (%)	
Present study	31	23/24 (95.8%)	22/26 (84.6%)	present
Haque et al, 2018 [4]	401	ND	ND	absent
Buchanan et al, 2008 [28]	52	ND	ND	absent
Middleton et al, 2000 [8]	38	ND	15/21 (71%)	absent
Lien et al, 2015 [33]	21	ND	ND	absent
Weidner et al, 1992 [7]	16	ND	ND	absent
Bentz et al, 1998 [48]	12	ND	9/11 (81.8%)	absent
Eusebi et al, 1992 [6]	10	ND	10/10 (100%)	absent
Weigelt et al, 2008 [20]	4	NA	NA	absent

AR, androgen receptor, GCDFP-15, Gross Cystic Disease Fluid Protein 15, ND, not done, NA, not available, PLC, pleomorphic lobular carcinoma

In conclusion, we reported rare case series of invasive PLC in Japan. We demonstrated that PLCs exhibit various surrogate molecular subtypes and triple-negative PLCs frequently express AR, indicating that molecular apocrine differentiation is observed in the LAR subtype. These findings implicate AR antagonists as a therapeutic option for patients with triple-negative PLCs.

## Supporting information

S1 File (XLS)
